# Empirical Mode Decomposition-Based Feature Extraction for Environmental Sound Classification

**DOI:** 10.3390/s22207717

**Published:** 2022-10-11

**Authors:** Ammar Ahmed, Youssef Serrestou, Kosai Raoof, Jean-François Diouris

**Affiliations:** 1 Laboratoire d’Acoustique de l’Université du Mans (LAUM), UMR 6613, Institut d’Acoustique-Graduate School (IA-GS), CNRS, Le Mans Université, 72085 Le Mans, France; 2 CNRS, IETR UMR 6164, Université de Nantes, 85000 La Roche-sur-Yon, France

**Keywords:** environment sound classification, acoustic signals, signal processing, time–frequency representations, empirical mode decomposition, intrinsic mode function, convolutional neural networks

## Abstract

In environment sound classification, log Mel band energies (MBEs) are considered as the most successful and commonly used features for classification. The underlying algorithm, fast Fourier transform (FFT), is valid under certain restrictions. In this study, we address these limitations of Fourier transform and propose a new method to extract log Mel band energies using amplitude modulation and frequency modulation. We present a comparative study between traditionally used log Mel band energy features extracted by Fourier transform and log Mel band energy features extracted by our new approach. This approach is based on extracting log Mel band energies from estimation of instantaneous frequency (IF) and instantaneous amplitude (IA), which are used to construct a spectrogram. The estimation of IA and IF is made by associating empirical mode decomposition (EMD) with the Teager–Kaiser energy operator (TKEO) and the discrete energy separation algorithm. Later, Mel filter bank is applied to the estimated spectrogram to generate EMD-TKEO-based MBEs, or simply, EMD-MBEs. In addition, we employ the EMD method to remove signal trends from the original signal and generate another type of MBE, called S-MBEs, using FFT and a Mel filter bank. Four different datasets were utilised and convolutional neural networks (CNN) were trained using features extracted from Fourier transform-based MBEs (FFT-MBEs), EMD-MBEs, and S-MBEs. In addition, CNNs were trained with an aggregation of all three feature extraction techniques and a combination of FFT-MBEs and EMD-MBEs. Individually, FFT-MBEs achieved higher accuracy compared to EMD-MBEs and S-MBEs. In general, the system trained with the combination of all three features performed slightly better compared to the system trained with the three features separately.

## 1. Introduction

Environment sound classification (ESC) has been receiving significant attention over the past couple of years. Environmental sounds range from traffic sounds, birds, rain, and sounds produced by human activity in houses, offices, cafes, and numerous other locations. In contrast to speech, environmental sounds are generated by various sources, including human and non-human activities. Humans mostly identify events or surroundings, in addition to vision, through the sounds present in the environment. The classification of such sounds provides a plethora of opportunities for improving human–machine interaction, thereby bimproving automation and security.

Neural networks have played a vital role in the growth of classification systems such as image classification systems and improved speech and environmental sound classification systems. Convolutional neural networks (CNN) are at the forefront of this change, along with recurrent neural networks (RNN) and long short-term memory (LSTM), which are still used in many systems [1,2,3,4,5,6,7,8]. In image classification, an image is used as feature. In speech and sound classification, an image form of the sound is provided through the time–frequency–energy information of the signal, namely, a spectrogram. Fourier transform is used extensively for spectrogram construction and feature extraction. It has dominated since the time of its inception and, consequently, the terms `spectrum’ and `spectrogram’ have become synonymous with Fourier transform of the data [9,10,11]. In classification systems, the Mel filter bank energies are extracted using a fast Fourier transform-based algorithm to generate Mel spectrograms. Whether these systems are trained from the scratch using time–frequency representation of sounds [6,12,13] or if transfer learning is used to retrain systems trained on images to perform sound classification [5,14,15,16], they employ Fourier transform for feature extraction. However, there are some crucial restrictions to performing Fourier spectral analysis, which makes Fourier transform valid under extremely general conditions [17,18]. To perform Fourier spectral analysis on a system, the system must be linear and the data must be ergodic and stationary; failing to meet these criteria will result in little physical sense. Sound is a time-varying signal whose frequency and energy changes depend on the source generating the sound, which implies that the assumptions of stationarity and ergodism may not be satisfied. In addition, the Fourier spectrum establishes global uniform harmonic components, resulting in the necessity of additional components to simulate data that is non-stationary and non-uniform globally. Consequently, it spreads the energy over a wide frequency range. To analyse data of a non-stationary nature in the time domain, numerous Fourier components are applied, causing energy diversion to a much wider frequency scale. Furthermore, Fourier spectral analysis utilises a priori-defined basis functions that require additional harmonic components to analyse deformed wave-profiles. Features based on short-time fast Fourier transform (STFT), introduced by Cooley and Tukey in 1965 [19], are predominately used in extracting frequency domain features [1,20,21,22,23]. The wavelet transform, which is a windowed Fourier transform in the time domain, provides the solution to the limitations of STFT. Wavelets [24] overcome limitations because the window is scaled in both time and frequency [25]. Wavelet analysis provides the solution for analysing non-stationary data. However, in wavelet transform, we still require an a priori-defined basis in terms of wavelet function, which makes wavelet analysis non-adaptive in nature. The most commonly used Morlet wavelet function is based on Fourier and suffers from the same shortcomings as Fourier analysis [18,26,27].

Due to ubiquitous usage of Fourier spectral analysis, the notions of instantaneous frequency (IF) and instantaneous amplitude (IA) are relatively less accepted [18]. Traditionally, the frequency is defined with the sine and cosine functions as basis functions spanning the whole data length with constant amplitude. According to this approach, the instantaneous frequency also must be defined on either the cosine or sine basis function. As result, it would be compulsory to have a one complete oscillation. This approach would make no sense for a non-stationary signal that changes from time to time. In real life, most systems are non-linear and operate or generate non-stationary data [28,29]. In order to cater to the non-linear and non-stationary nature of signals, a novel method of decomposing temporal signals to analyse non-stationary and non-linear time series data and processes called empirical mode decomposition was introduced by Huang [18]. This decomposition is adaptive and highly efficient. This method decomposes the signal into a finite number of oscillatory units called intrinsic mode functions (IMFs). These modes are extracted based on characteristics of local time series data with zero mean with symmetric AM–FM components. The decomposition of the signal is highly adaptive and is based on the direct extraction of energy with local time scales. Using the Teager–Kaiser energy operator (TKEO), we can extract instantaneous frequency and amplitude from the IMFs, thus, allowing us to locate any event on a time scale and a frequency scale. The IMFs serve as the basis in this case and are calculated for every signal rather than being defined a priori. The EMD combined with the TKEO method provides the estimation of instantaneous amplitude (IA) and instantaneous frequency (IF) for any non-stationary signal without defining an a priori basis function; this method generates the basis function dynamically for each signal. The advantage of this method is that it uses instantaneous amplitude and frequency analysis and does not rely on any basis functions compared to other techniques to generate time frequency-based features, generating instantly rather than requiring a minimum number of samples to perform analysis. In addition, this preliminary work adds another path for future development and applications of IA and IF in different domains where time frequency analysis is required. The EMD method has been used in speech recognition systems [30,31,32] and human emotion recognition system [33]. The EMD method has also been used to perform classification of respiratory sound in conjunction with FFT to extract features [34]. The EMD extracts IMFs and later selects the best IMF based on the entropy parameter. Gamma tone cepstral coefficients (GTCC) are extracted using Fourier transform and discrete cosine transform. However, in this approach, the underlying issue due to Fourier transform persists.

In ESC system, we are interested in the features extraction stage. The system relies heavily on the type of the features to learn sound events. In this paper, we introduce the use of empirical mode decomposition along with the Teager–Kaiser energy tracking operator to estimate instantaneous frequency and amplitude, which are used to construct features in terms of spectrogram for classification using neural networks. We apply the most commonly used Mel filter banks for the spectrogram. In this paper we introduced the novel Mel filter based on a spectrogram generated through IA and IF. The EMD method decomposes the signal into several mono-component IMFs; on each of the IMF, TKEO and DESA methods are applied to obtain the IA and IF information of the signal. We call this Mel spectrogram obtained through EMD and TKEO the empirical mode decomposition Mel filter bank energies (EMD-MBE). We also introduce SMBE, in which we remove the signal trend from the signal using the EMD method. We compared our proposed features with fast Fourier-based Mel filter bank energies (FFT-MBE) on four ESC data-sets. We propose an aggregation of all three features, which results in improvement of accuracy over traditional FFT-based log Mel filter bank energies.

The rest of the paper is organised as follows. Section 2 explains the EMD and TKEO methods in detail. Section 3 demonstrates feature extraction process using FFT and EMD-TKEO methods. In Section 4, the experimental setup is described, followed by Section 5 discussing the results obtained. Section 2 presents the conclusion.

## 2. Empirical Mode Decomposition-Teager–Kaiser Energy Operator (EMD-TKEO) Method

**Figure 1:**
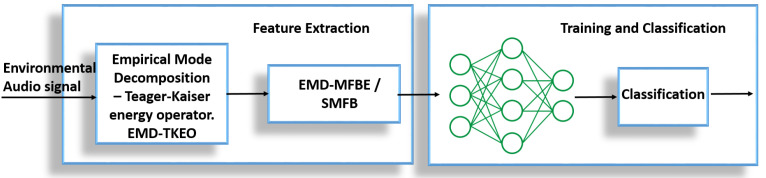
Block diagram of proposed system—feature extraction using EMD-TKEO method and classification using neural networks.

We use the empirical mode decomposition method to decompose the environmental sound into its intrinsic mode functions (IMFs), as described in the next section. The combination of EMD decomposition and TKEO is used to estimate instantaneous amplitude (IA) and instantaneous frequency (IF). Afterwards, a Mel filter bank is applied to obtain Mel filter bank energies (MBE). These features are then used to train machine learning algorithms. The proposed system is depicted in the Figure 1.

In this paper, we extend the work of P. Maragos [35,36] from the application of speech and underwater acoustic signals to extracting AM–FM modulation information from environment sound signals. In [35], the authors defined the real valued signal with combined AM and FM structure as:

(1) $$r_{i}\left(t\right)=Re\left(a_{i}\left(t\right)*exp\left[j\phi_{i}\left(t\right)\right]\right)$$

This expression can be utilised to formulate a signal as [37]:

(2) $$x\left(t\right)=\sum_{i=1}^{N}r_{i}\left(t\right)+rest_{(}t)$$

and

(3) $$f_{i}\left(t\right)=\frac{1}{2\pi }\frac{d\phi_{i}\left(t\right)}{dt}$$

where rest(t) is the last component containing very low frequency information, which could be neglected from the original signal. Re represents the real part, $\phi_{i}\left(t\right)$ is the phase, and $a_{i}\left(t\right)$ and $f_{i}\left(t\right)$ are instantaneous amplitude and instantaneous frequencies respectively of the *i*th IMF.

### 2.1. Empirical Mode Decomposition

EMD is a method of a decomposing non-stationary signal into a collection of mono-component AM–FM signals. These mono-component signals are referred to as intrinsic mode functions (IMFs). The extraction of the IMFs follows an envelope subtraction process and linear combination of all the IMF-extracted results into the original signal. The signal is decomposed in the time domain, hence preserving the time-varying frequency and amplitude of the signal. As compared to Fourier transform, EMD does not require an a priori-defined basis function for the computation of IMFs. Fourier transform uses harmonic components of signal, whereas EMD is based on the oscillation present in the signal. The oscillatory decomposition is defined by the sifting process. The signal is examined for local maxima and minima. Using the information of local maxima and minima, the upper envelope and lower envelope are determined via cubic spline. The mean envelope is generated using the upper and lower envelopes, which represents the trend of the signal. This mean envelope is subtracted from the original signal to create an IMF candidate. Before counting this candidate as an actual IMF, a test is conducted, i.e., if the number of zero crossings and the number of extrema differs by no more than one. If the candidate satisfies the criteria, it is counted as an IMF and the counter is incremented; otherwise, the counter is set to zero. Verification is conducted to check if the candidates meet the criteria of IMF for each IMF generated. If the criteria are not fulfilled, the sifting process is applied again until the conditions are matched. The IMF obtained is then stored and is subtracted from the original signal to start a new sifting process for another IMF. The method is repeated until the signal is deconstructed to a level that it contains no more than two extrema [18].

### 2.2. Sifting Process for IMFs

The EMD method could be defined in simplest terms as a filter that sifts through the signal and breaks it down into a mono-component signal, defined above as IMFs. A function is defined as an intrinsic mode function when it satisfies the following criteria:

1 The number of extrema (maxima and minima) in a signal must be equal to the zero-crossing number or differ at most by one;
2 The mean of the envelopes obtained through local maxima and local minima must be equal to zero at all times.

The IMFs are obtained through a process known as the sifting process, which is described in Algorithm 1 [33]:
**Algorithm 1:** Sifting process for intrinsic mode functionsInput: a sound event signalOutput: collection of IMFs
 1Compute all local extrema in the signal x(t): local maxima and local minima;2Construct the upper envelope $E_{u}\left(t\right)$ and lower envelope $E_{l}\left(t\right)$ by joining the local maxima and local minima with a cubic spline on the given signal x(t);3Calculate the mean of the envelopes m(t) = ($E_{u}\left(t\right)$ + $E_{l}\left(t\right)$)/2;4Subtract the mean from the original signal x(t), then obtain a new data sequence r(t) from which the low frequency is deleted r(t) = x(t) - m(t);5Repeat steps 1−4 until r(t) is an IMF (satisfying the two conditions above);6Subtract this IMF r(t) from the original signal x(t): res(t) = x(t) - r(t);7Repeat steps 1−6 until the residual signal res(t) is obtained that does not meet the above mentioned conditions of an IMF, resulting in all IMFs $r_{1}\left(t\right)$, $r_{2}\left(t\right)$,…, $r_{N}\left(t\right)$ of the signal x(t).

The number of IMFs extracted from a particular signal depends on two factors.

1 The process terminates when the res(t); the last IMF, is either a monotonic function or function with only one extremum.
2 The number of IMFs is subjected to stopping criteria, where the user terminates the sifting process after a particular number of IMFs have been created.

In the first case, the output of the EMD sifting process delivers *N* IMFs $r_{1}\left(t\right)$, $r_{2}\left(t\right)$,…, $r_{N}\left(t\right)$ along with the residual signal res(t) of the original signal x(t). x(t) can be presented as a linear combination of all the IMFs and res(t).

(4) $$x\left(t\right)=\sum_{i=1}^{N}r_{i}\left(t\right)+rest_{N}\left(t\right)$$

**Figure 2:**
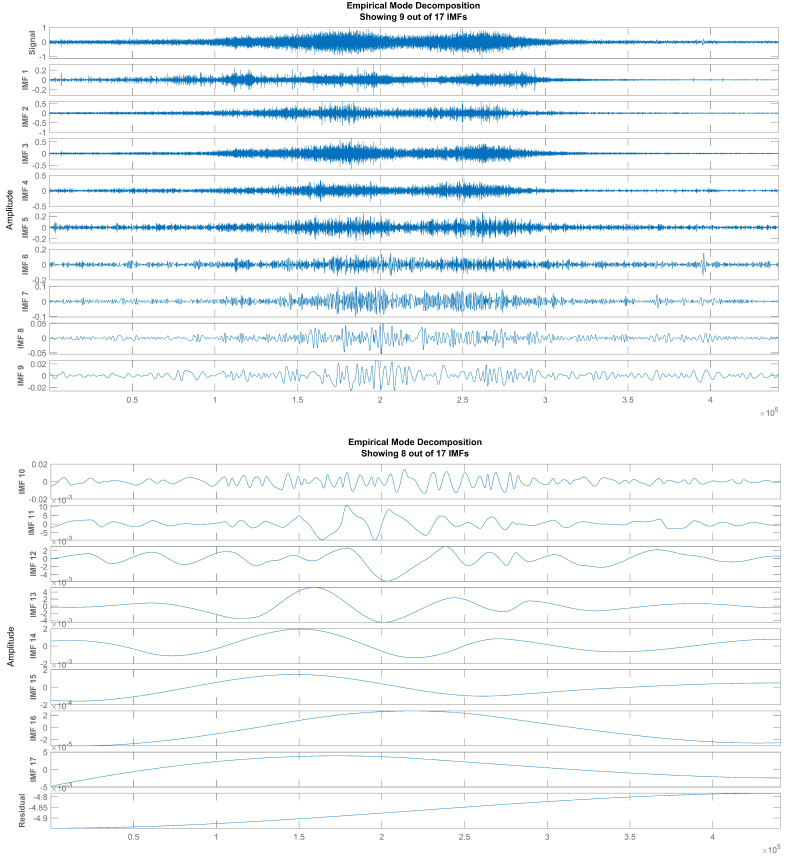
Empirical mode decomposition and intrinsic mode function extraction.

With this method, the signal x(t) is decomposed empirically into a finite number of functions. The IMFs of an audio of car passing is show in Figure 2. Each IMF can be used separately to obtain instantaneous Frequency (IF) and instantaneous amplitude (IA) for sound event detection systems, explained in the next section.

In the case of early stopping, the original signal cannot be reconstructed, as some information is discarded deliberately. However, in some cases, it could be used to remove low frequency components from the parent signal. In [38], the authors used the first five IMFs, on the basis that those IMFs gave an ample amount of information about energy and pitch in their study.

### 2.3. Teager–Kaiser Energy Operator (TKEO)

The energy separation algorithm (ESA) is applied to extract the IA and IF information from the signal, as standalone IMFs do not provide meaningful information about IA and IF. The EMD method takes a multi-component signal and provides us with IMFs that are mono-component. Introduced by J.F. Kaiser [39], the TKEO, an energy tracking operator used with an energy separation algorithm, computes these IA and IF features without using integrals, as in the Hilbert transform and Fourier Transform. Rather it is completely comprised of differentiation. The property of differentiation gives the TKEO the advantage of good localisation [40]. It becomes more natural to use the TKEO for local estimation of IA and IF functions. The TKEO is a non-linear operator that computes the energy of the signal as a product of the square of the amplitude and frequency of the signal, given as in [41]:

(5) $$\Psi \left[r_{i}\left(t\right)\right]={\left[{\dot{r}}_{i}\left(t\right)\right]}^{2}-r_{i}\left(t\right){\ddot{r}}_{i}\left(t\right)$$

where ${\dot{r}}_{i}\left(t\right)$ and ${\ddot{r}}_{i}\left(t\right)$ are the first and second order derivatives of $r_{i}\left(t\right)$. For a discrete time signal $r_{i}\left(n\right)$, Equation (5) can be written as [42]:

(6) $$\Psi \left[r_{i}\left(n\right)\right]=r_{i}^{2}\left(t\right)-r_{i}\left(n+1\right)r_{i}\left(n-1\right)$$

The instantaneous features are extracted by applying ESA in a discrete form to the signals. The discrete energy separation algorithm (DESA) [41] provides us with IA and IF. We used DESA-1 in this study, given as:

y(n)=x(n)−x(n−1)

(7) $$f\left(n\right)\approx arccos\left(1-\frac{\Psi [y(n)]+\Psi [y(n+1)]}{4\Psi [y(n)]}\right)$$

(8) $$\left|a\left(n\right)\right|\approx \frac{2\Psi [x(n)]}{\sqrt{\Psi [x(n+1)-x(n-1)]}}$$

**Figure 3:**
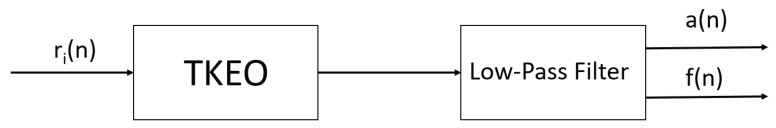
Block diagram of smoothing output of the energy operator.

Here, x(n) is a mono-component signal. The DESA-1 algorithm should be applied on mono-component signals only. In [36], authors proposed the use of low pass filter to smooth the output of the energy tracking operator. They found that high frequency error component was introduced by the energy operator. To eliminate this issue, a seven-point linear binomial low-pass smoothing filter with impulse response (1,6,15,20,15,6,1) is applied after TKEO as shown in Figure 3 [33,36].

## 3. Feature Extraction

### 3.1. Mel Band Energies

The general Mel band energies (MBEs) are computed through discrete Fourier transform as follows. Let x(n) be a discrete audio signal having sampling rate $f_{s}$. It is divided into *P* frames, each of length *N* samples with N/2 overlapping samples, such that $\left\{{\vec{x}}_{1}\left[n\right],{\vec{x}}_{2}\left[n\right],...,{\vec{x}}_{p}\left[n\right],...,{\vec{x}}_{P}\left[n\right]\right\}$, where ${\vec{x}}_{p}\left[n\right]$ represents the pth frame of the signal x[n] and is given as:

(9) $${\vec{x}}_{p}\left[n\right]={\left\{x\left[p*\left(\frac{N}{2}-1\right)+i\right]\right\}}_{i=0}^{N-1}$$

The input signal x[n] can be represented as a matrix of size N×P as $X=[{\vec{x}}_{1},{\vec{x}}_{2},...,{\vec{x}}_{p},...,{\vec{x}}_{P}]$. When calculating DFT, the signal for each ${\vec{x}}_{p}$, one assumes that the signal is repeated infinitely, which introduces an issue of spectral leakage. To avoid spectral leakage, the Hanning window is applied.

(10) $$w_{[}n]=0.5*\left(1+\cos\left(\frac{2\pi n}{N}\right)\right)$$

and the discrete Fourier transform of the signal is given as:

(11) $$X_{p}\left(k\right)=\sum_{n=0}^{N-1}x_{p}\left[n\right]w\left[n\right]\exp^{-j\frac{2\pi kn}{N}}$$

**Figure 4:**
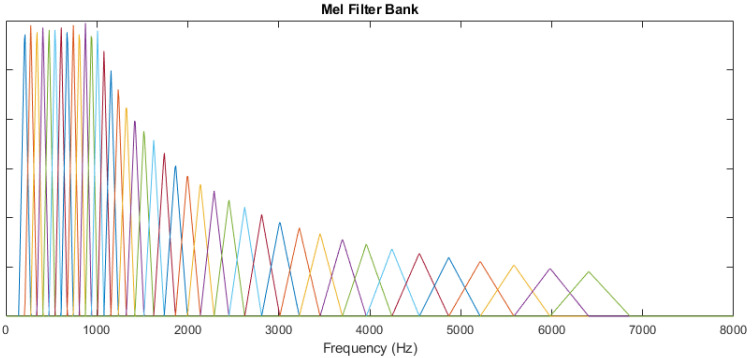
Mel triangular filter bank.

Here, k=0,1,2,...,N−1, where *N* represents the number of points used by FFT for a particular frame $x_{p}$. Using the sampling rate $f_{s}$ of the input signal, the corresponding frequency can be computed using the frequency bin as $l_{f}\left(k\right)=kf_{s}/N$ and the frequency resolution can be computed as $f_{r}es=l_{f}\left(k+1\right)-l_{f}\left(k\right)$. The DFT of the $p^{th}$ frame $x_{p}$ can be represented as ${\vec{X}}_{p}={\left[X_{p}\left(0\right),X_{p}\left(1\right),X_{p}\left(2\right),\ldots ,X_{p}\left(N-1\right)\right]}^{T}$; similarly, for the complete signal x[n]m we obtain $X=[{\vec{X}}_{1},{\vec{X}}_{2},...,{\vec{X}}_{P}]$. Here, the *X* matrix has the dimensions N×P and is defined as a short-time Fourier transform. The magnitude spectrum of the signal is obtained by taking the modulus of *X*. The magnitude spectrum is warped according to the Mel scale to obtain human ear-like properties. The Mel frequency $\left(\phi_{f}\right)$ and the linear frequency $l_{f}$ are defined by the relation $\phi_{f}=2595*log_{10}\left(1+\frac{l_{f}}{700}\right)$. Mel filter banks, which are comprised of overlapping triangular filters defined by their centre frequencies $l_{f_{c}}\left(m\right)$, are used to segment the spectrum *X* depending on the band number *m*. The Mel filter bank is shown in Figure 4.

The three parameters that define Mel filter banks are:

- Number of Mel filters, F;
- Minimum frequency, $l_{f_{min}}$;
- Maximum frequency, $l_{f_{max}}$.

Using the minimum and maximum frequencies and the number of Mel filters, constant frequency resolution is calculated using the relation $\delta \phi_{f}=\left(\phi_{f_{max}}-\phi_{f_{min}}\right)/\left(F+1\right)$, where $\phi_{f_{max}}$ and $\phi_{f_{min}}$ are frequencies on the Mel scale defined by the corresponding linear frequencies $l_{f_{max}}$ and $l_{f_{min}}$, respectively. The centre frequencies on the Mel scale are obtained through $\phi_{f_{c}}\left(m\right)=m.\delta \phi$ and m∈{1,2,3,…,F}. Similarly, we can inverse the relation to obtain centre frequencies in the linear frequency in Hz as $l_{f_{c}}\left(m\right)=700\left(10^{\phi_{f_{c}}\left(m\right)/2595}-1\right)$. The resulting Mel filter bank matrix M (m, k) of size F×N is given by:

$$M\left(m,k\right)=\left\{\begin{array}{cc} 0 & \mathrm{for}\;l_{f}\left(k\right)<l_{f_{c}}\left(m-1\right)\\ \frac{l_{f}\left(k\right)-l_{f_{c}}\left(m-1\right)}{l_{f_{c}}\left(m\right)-l_{f_{c}}\left(m-1\right)} & \mathrm{for}\;l_{f_{c}}\left(m-1\right)\leq l_{f}\left(k\right)<l_{f_{c}}\left(m\right)\\ \frac{l_{f}\left(k\right)-l_{f_{c}}\left(m+1\right)}{l_{f_{c}}\left(m\right)-l_{f_{c}}\left(m+1\right)} & \mathrm{for}\;l_{f_{c}}\left(m\right)\leq l_{f}\left(k\right)<l_{f_{c}}\left(m+1\right)\\ 0 & \mathrm{for}\;l_{f}\left(k\right)>l_{f_{c}}\left(m+1\right) \end{array}\right.$$

To obtain Mel filter bank energies, we multiply the DFT matrix $X_{p}\left(k\right)$ by the Mel filter bank matrix M(m,k). A logarithm is applied to obtain log Mel band energies of the size F×P, as given in.

(12) $$S_{p}\left(m,k\right)=log\left\{\sum_{k=0}^{N-1}M\left(m,k\right)*\left|X_{p}\left(k\right)\right|\right\}$$

### 3.2. EMD-Mel Band Energies

**Figure 5:**
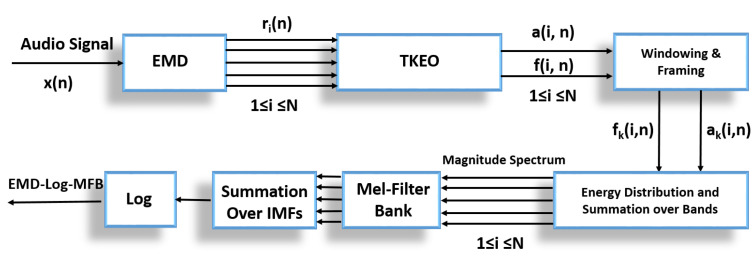
Empirical mode decomposition-based Mel filter bank energies extraction block diagram.

In this method, AM and FM components are used to construct magnitude spectrum; later a Mel filter bank is applied to obtain Mel band energies. Figure 5 demonstrates the process of obtaining MBEs; in this case, we call these features empirical mode decomposition-based Mel filter bank energies (EMD-MBE). The first step is the decomposition of the signal into its components using EMD. These distinct, adaptive decomposed components are known as intrinsic mode functions (IMFs). For each distinct IMF, instantaneous amplitude a(i,n) and instantaneous frequency f(i,n) are obtained through the energy tracking operator and energy separation algorithm, where $i=1,2\ldots N_{IMFs}$ and $n=1,2,3,\ldots ,N_{b}$, and $N_{b}$ represents the number of samples in the input signal. In this study, we used the Teager–Kaiser energy tracking operator (TKEO) and the discrete energy separation algorithm (DESA-1). The TKEO and DESA compute the IA and IF over the complete length of the IMF. The TKEO and DESA algorithms in [36,43,44] have shown estimations of IA and IF with an error less than $10^{-3}$. The framing /windowing function is applied later to the IA and IF obtained from the TKEO. In the next stage, we apply the Hanning windowing function to obtain short overlapping frames of instantaneous frequency $f_{p}\left(i,n_{p}\right)$ and instantaneous amplitude $a_{p}\left(i,n_{p}\right)$. Afterwards, to obtain the magnitude spectrum, we use the definition provided by [37]. The authors defined the Hilbert Huang Transform as a generalised Fourier Transform and defined the spectrum using the Hilbert spectrum, which is derived from the time–frequency distribution of the instantaneous energy envelope, which is the square magnitude of the amplitude envelope. In this study, we used the instantaneous energy envelope $\left(|a\left(i,n\right){|}^{2}\right)$ and summed them over large number of frequency bands, as compared to the Hilbert spectrum [33,37]. This enables us to distribute the energy over a large number of frequencies and obtain higher frequency resolution. To derive Mel band energies, we summed the energies similarly to the number of frequency bands defined in the previous section. The relation is defined as (13):

(13) $$X_{p}\left(f\right)=\sum_{i=1}^{N_{IMFs}}\sum_{n=1}^{N}{\left|a\left(i,n\right)\right|}^{2}1_{B_{k}}\left(f\right)$$

where *i* is a single IMF, *N* represents the number of samples in the frame, and $B_{j}$ represents the particular sub-band defined as $B_{k}=\left[l_{f}\left(k\right),l_{f}\left(k+1\right)\right]$, where k=1,2,3,...N−1 and $l_{f}\left(k\right)=kf_{s}/N$. The indicator function of set Ω is given as:

$$1_{\Omega }\left(a\right)=\left\{\begin{array}{cc} 0 & \mathrm{if}\;a\in \Omega \\ 1 & \mathrm{if}\;a\notin \Omega \end{array}\right.$$

After using (13), we obtain a matrix of size N×P. This matrix is multiplied, similarly to Equation (12), by the Mel filter bank matrix M(m,k) of shape F×N. The resulting matrix generated will have the shape F×P. Taking the log, we obtain log Mel band energies.

### 3.3. S-MBE

**Figure 6:**
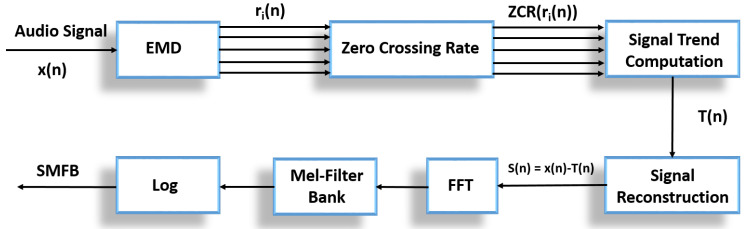
SMBE feature extraction block diagram.

Sounds produced in any environment are composed of complex and random changes, and the presence of a signal trend causes a negative effect in frequency domain power spectral analysis or time domain correlational analysis, which could result in the loss of information in the low frequency spectrum. To counter such problems, we apply a method of extracting Mel filter bank energies (MBEs) by removing the signal trend, calling these S-MBEs. In the literature, researchers have used a version of SMFCC for emotion recognition [45]. We present a different version that extracts the feature before applying discrete cosine transform (DCT) to the MBEs. The complete process of extracting S-MBEs is presented in Figure 6. In this method, EMD performs the decomposition on the signal and extracts the signal trend information from the IMFs. The signal trend is denoted by T[n] and is computed by Equation (14) [45]:

(14) $$T\left(n\right)=\sum_{i}^{N_{IMFs}}r_{i}\left(n\right)$$

The signal trend is removed from the input signal by applying the zero-crossing rate (ZCR) detection method. T[n] is defined as the sum of all IMFs that satisfy the following condition Equation (15):

(15) $$\frac{R_{ri}}{R_{r1}}<0.01\;\left(i=2,\ldots ,n\right)$$

where R represent the zero-crossing rate. Afterwards, the reconstructed signal S[n] is obtained by removing the signal trend T[n] from the input signal.

(16) S[n]=x[n]−T[n]

Finally, the MBEs are calculated using FFT and Mel filter banks on the reconstructed signal, as shown in Figure 6.

### 3.4. Features

**Figure 7:**
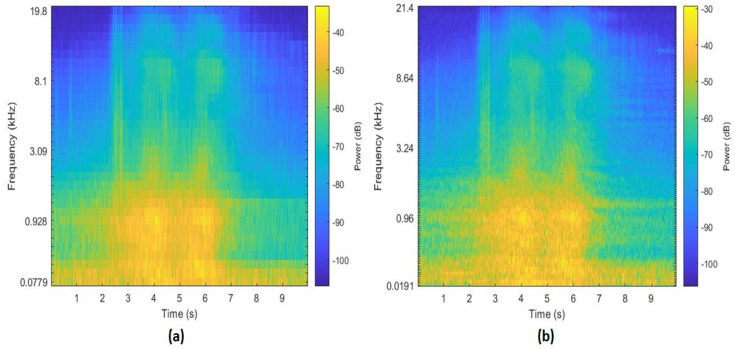
Spectrograms extracted from a 10 sec audio file of a car passing. (**a**) FFT-MFB spectrogram. (**b**) S-MFB spectrogram.

**Figure 8:**
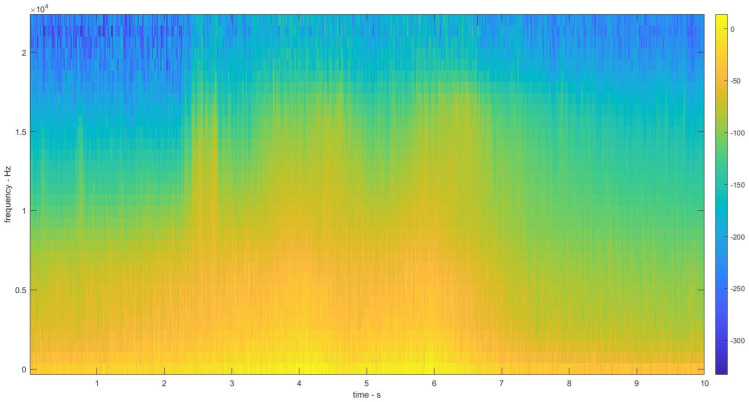
Empirical mode decomposition-based Mel spectrogram from a 10 sec audio file of a car passing.

The Mel spectrograms from the aforementioned mentioned techniques are extracted and are depicted in Figure 7 and Figure 8. The FFT based Mel spectrogram is shown in Figure 7a along with S-MBE in Figure 7b. The EMD based Mel spectrogram is depicted in Figure 8.

## 4. Experimental Setup

### 4.1. Databases

The development of an ESC system relies heavily on the database. Sound classification is a vast topic and contains many categories such as acoustic scene classification, sound event classification, environmental sound classification, and many more. We utilised databases comprised of acoustic scenes, sound events, and environmental sounds for classification. The databases are explained below.

#### 4.1.1. Acoustic Scene Classification Dataset

Detection of acoustic scene has been considered as a complex problem by the research community for several years, and various efforts have been dedicated to solving this issue. Acoustic scenes contain acoustic events in a particular environment such as metro station, airports, train stations, etc. Classifying these categories becomes complex due to the similar nature of the sound events occurring in those environments. To solve this issue, Detection and Classification of Acoustic Scenes and Events (DCASE) provided a dataset that contains audio recordings of 10 different categories: airport, indoor shopping mall, metro station, pedestrian street, public square, street with medium level of traffic, travelling by a train, travelling by a bus, travelling by an underground metro, and urban park (TUT Urban Acoustic Scenes 2018 dataset).

#### 4.1.2. Low-Complexity Acoustic Scene Classification Dataset

This dataset is provided by the DCASE community [46] and contains three categories. The dataset comprises recordings from 12 European cities in 10 distinct acoustic scenes. The 10 different categories are then divided into three separate categories as follows:

- Indoor scenes—indoor: airport, indoor shopping mall, and metro station;
- Outdoor scenes—pedestrian street, public square, a street with a medium level of traffic, and urban park;
- Transportation-related scenes—traveling by bus, traveling by tram, traveling by underground metro.

The audio signal is recorded at 48kHz and in 24-bit in binaural format using only one recording device. The dataset is divided into two categories: the development set and the evaluation set. Due to the unavailability of the labels of the evaluation set, the system was evaluated on the development set only. The development set contains 40 h of audio recordings divided into a training set and a test set. Each audio file is 10 s long. The baseline system [47] is evaluated using the development set by log Mel filter bank energy features.

#### 4.1.3. Urbansound8k

Urbansound8k is a dataset containing 10 different classes and 8732 short-duration (less than or equal to 4 s) files [48,49]. The collection is composed of environmental sounds such as air conditioner, car horn, playing children, dog bark, drilling, engine idling, gun shot, jackhammer, siren, and street music. Recordings are available in 10-fold cross validation and recorded at 22.05 KHz sampling frequency.

#### 4.1.4. Custom Database

The audio recordings are collected from FreeSound [50] from several contributors. Each recording was registered by a different publisher and with different locations, lengths, equipment, and sampling rates. The recordings are gathered for four categories, i.e., rain, wind, car passing, and human walk. The recordings sampling rates were from 44,100 Hz to 96,000 Hz. The database was processed to obtain uniform characteristics. Ten-second audio files were extracted with a sampling rate of 441,000 Hz, resulting in 750 files of 10-s length with a total duration of 125 min for each recording [51].

### 4.2. Classification Model

We used convolutional neural networks (CNNs) in this study. CNNs have been widely used with Mel band energies for classification of environmental sounds. For the Acoustic Scene Classification Dataset and the Low-Complexity Acoustic Scene Classification Dataset, we used baseline [46] CNN1 model. All the parameters were selected according to the baseline model mentioned by the authors in order to evaluate the same system using different feature inputs. For Urbansound8K and the custom dataset, we used the CNN2 model [51] given in Table 1.

In this study, we made comparison between two feature extraction techniques. We have proposed an EMD-based feature extraction technique compared to FFT-based feature extraction technique. In order to compare the performances of both methods, we employed baseline systems. We used baseline systems since they are built on the simple extraction of Mel band energies features, and no additional pre- or post-processing is applied during the training and testing of the systems. The systems that reached highest score of accuracy on the evaluation of acoustic scene classification datasets [52] use an ensemble of features based on adaptive temporal division and classify using a VGGish based neural network. In addition, the score on the development dataset is not published. The leading system on the development dataset [53] uses MFCC features with I-vector backend processing with a fusion of CNNs and I-vectors to make predictions. Similarly, for the low-complexity acoustic scene classification dataset, the leading system uses resnet with a receptive field. For Urbansound8k, different systems are proposed [16,54]. These systems use feature pre-processing and post processing, transfer learning, and other methods to enhance the accuracy of the system. Compared to the state-of-the-art systems, which utilize different systems and employ different pre and post processing methods, we followed the path of baseline systems. To compare against FFT-based Mel band energies, we proposed EMD-based Mel band energies. This allows us to evaluate the performance of both features on the same system and with same parameters without any pre- or post- processing of the feature. The specifications of the systems used are described below.

- Log-scaled Mel band energies were extracted for every dataset. For the Acoustic Scene Classification Dataset and Low-Complexity Acoustic Scene Classification Dataset, we extracted 40 Mel bands using an analysis frame of 40 ms with a 50% overlap. Similarly, the EMD-Log Mel band energies and log scaled S-MBEs were calculated for both datasets with similar characteristics, resulting in similar shape. The input shape is 40×500, trained for 200 epochs with a mini batch size of 16 and data shuffling between epochs. An Adam optimizer [55] is used for optimisation, with a learning rate of 0.001. Model performance is checked after each epoch on the validation set, and the best performing is chosen. The system was trained for 200 epochs with the Adam optimizer, with an initial learning rate of 0.001.
- For Urbansound8k, the log Mel band energies, EMD-log Mel band energies, and log scaled S-MBEs were extracted with 60 Mel bands; a window size of 1024 samples with a hop length of 512 samples is used. The input size for the CNN was 60×41 and silent segments were discarded. The Urbansound dataset was trained using 10-fold cross validation. The network was trained for 300 epochs with the Adagrad optimizer [56].
- For the custom database, the log Mel band energies, EMD-log Mel band energies, and log scaled S-MBEs were extracted with 128 Mel bands with 50% overlap. The custom dataset was trained using seven-fold cross validation. The system was trained for 200 epochs with Adagrad optimizer, with an initial learning rate of 0.001.
- To evaluate the experimental results, this paper uses classification accuracy as a metric:
  $$Accuracy=\frac{TP+TN}{TP+TN+FP+FN}$$
  where TN and TP are defined as the number of negative and positive examples that are classified successfully, respectively. FN and FP are the number of misclassified positive and negative examples, respectively. The evaluation metric is chosen according to the baseline system [46] to perform comparisons between the feature extraction methods under the same evaluation metrics.

## 5. Results and Discussion

We trained the convolutional neural networks with the parameters given in the previous section. The model was evaluated using a test set and average classification accuracy is computed. First, we trained the models using only one feature at a time; for each feature, the system was trained and evaluated. Afterwards, we combined the time–frequency analysis techniques. In the first case, we combined FFT-MBEs and EMD-MBEs. The model was trained and evaluated by aggregating these two features. Later, we combined the two proposed features extracted using EMD-MBEs and SMBEs with traditional FFT-MBEs. Similarly, we trained and evaluated the models by aggregating all the features.

The average classification accuracy of each database with different features are presented in Table 2. The class-wise average classification accuracies for the Acoustic Scene Classification Dataset with FFT-based log MBE, EMD-based log MB, log SMBE, and a combination of these features are presented in Table 3. Similarly, for the Low-Complexity Dataset, Urbansound8k, and the custom database, class-wise mean classification accuracies are presented in Table 4, Table 5, and Table 6, respectively.

It is evident that the FFT-based log MBEs performed better than EMD-based log MBEs and log S-MBEs for every database. However, the combination of all the features improves performance of the system with respect to single FFT-based log MBE-based features in some cases. The EMD-MFB method outperforms FFT-MFB in some categories, as shown in the Table 2, Table 3, Table 4, Table 5 and Table 6. The CNNs were able to perform better inferences for some categories than others. It is, however, uncertain what led the CNNs to better learn features of one method over another, and of one category over another. There are no evaluation methods available in the research domain to understand what leads to better performance of CNNs. In EMD-MFB, for each sample of monotonic IMF in the time domain, we obtained an equivalent instantaneous amplitude IA and instantaneous frequency IF component. To obtain a similar shape as that for FFT-MBE, we performed framing on the training sample, and later we performed summation over frequency bands. The frame consists of IA and IF samples. The total number of frequency components in a single frame of EMD-MFBE for the original signal is defined by the number of samples in the window summed over frequency bands and the number of IMFs extracted. Contrary to this, in FFT, the windowing function is applied directly to the input signal and frequency and energy components are computed over this window. This method may introduce spurious higher harmonics in the result, as mentioned earlier. The FFT-MBE is smoother and contains more components per frame, whereas EMD-MBE is limited by the number of IMFs and number of samples in the window.

Furthermore, empirical mode decomposition suffers from mode mixing. Mode mixing of EMD is mainly caused by intermittence and noise. Sudden changes in the signal are one of the main causes of mode mixing, such as noise interference or a high frequency wave discrete distribution in the original signal, which results in the signal being a local high frequency signal, thus producing a local extreme value. The envelope generated by this local extreme value point jump phenomena results in the IMF not agreeing with the time scale and continues to the different frequency components in the original signals, which cannot be effectively separated according to the characteristics of time scale [57]. Mode mixing will affect the subsequent decomposition components; afterwards, the time–frequency distribution of following IMFs will be ambiguous and, eventually, the EMD decomposition process loses physical meaning [58,59]. Many researchers have studied this issue and there are several solutions have been given [60,61,62].

The EMD-MFB method requires more computational resources and time to extract features as compared FFT-based MFB. One evident reason is the calculation of IMFs during the decomposition of the signal. Secondly, the programming methods for the calculation of FFT have been highly studied, which resulted in the current algorithm of Cooley and Tukey [19]. Similar efforts could be made in future for the EMD method in order to reduce the computational overhead.

## 6. Conclusions

The main objective of this paper was to introduce an adaptive time–frequency analysis method for an audio signal and perform a comparative analysis with traditionally used time–frequency analysis method. These methods were evaluated based on their performance as features in an environmental sound classification system. The traditionally used method, Fourier transform, is valid under some general conditions and relies on an a priori-defined basis. An adaptive method for signal decomposition into multiple components introduced by Huang et al., empirical mode decomposition (EMD), is applied to obtain intrinsic mode function (IMFs) as components. A discrete energy separation algorithm, the Teager–Kaiser energy operator (TKEO), is applied on each IMF individually to obtain instantaneous amplitude (IA) and instantaneous frequency (IF) on a local time scale. Afterward, a windowing function is applied to generate spectrograms, which are summed together. Later, a Mel filter bank is applied to generate log Mel band energies. We also proposed S-MBEs in this paper, which use EMD to compute the signal trend, which is subsequently removed from the original signal; later, log Mel band energies are computed using Fourier transform (FFT). The features extracted from the proposed method estimated the change of frequencies with respect to time similarly to traditional method with different intensities. This could be attributed to the fact the EMD-TKEO method estimated the IA and IF, which were summed together with a fixed window size to match the dimensions of Mel filter banks. We compared the performance of features extracted with the proposed methods with features extracted from fast Fourier transform-based log Mel band energies. Two different CNN systems were employed in this study to evaluate the feature performance on four different databases. The results demonstrate that the EMD-based Mel band energies (EMD-MBEs)’s performance lagged behind FFT-based Mel band energies (FFT-MBEs). S-MBE performed the worst among the three features under evaluation for every database. The aggregation of all three features resulted in an improvement of accuracy over FFT-MBEs. The improvement reflects the fact that EMD-MBEs performed better for some classes than FFT-MBEs, and the combination of these methods improved the overall result. The analysis of the low performance of the proposed method reveals that in the estimation of time–frequency representation, the resolution is limited by the number of IMFs and window size. Furthermore, during the process of decomposition of the signal into IMFs, the EMD method suffers from the mode mixing problem, which degrades the quality of features extracted. In the future, different EMD methods will be under consideration to obtain estimation of features with similar performance to FFT-based features.

## Figures and Tables

**Table 1:** Convolutional neural network model specification.

Layers	Model Specifications	
	*CNN1*	*CNN2*
Layer 1	Conv2D (32,(7,7))	Conv2D (64,(4,4) )
	Relu, strides = 1	tanh, strides = 1
	MaxPool2D (5, 5)	MaxPool2D (2, 2), stride = 2
	Dropout (0.3)	
	Batch normalisation	
Layer 2	Conv2D (64,(7,7))	Conv2D (32,(4,4) )
	Relu, strides = 1	tanh, strides = 1
	MaxPool2D (4, 100)	MaxPool2D (2, 2), stride = 2
	Dropout (0.3)	Dropout (0.2)
	Batch normalisation	
Layer 3	-	Conv2D (16,(4,4) )
		tanh, strides = 1
		MaxPool2D (2, 2), stride = 2
		Dropout (0.2)
Layer 4	Dense (100)	2 X Dense (400)
	Activation = relu	Activation = tanh
	Dropout = 0.3	
Layer 5	-	Dense (300)
		Activation = tanh
		Dropout = 0.2
Layer 6	Dense (classes, softmax)	

**Table 2:** System performance comparison.

Features	Accuracy per Database			
	ASC Dataset	Low Complexity ASC	Urbansound8k	Custom
FFT	54.77%	84.18%	63.36%	75.61%
EMD	52.5%	79.74%	54.64%	75.05%
SMB	48.25%	79.55%	52.41%	71.93%
FFT+EMD	56.08%	84.49%	63.70%	78.87%
FFT+EMD+SMB	57.78%	84.83%	62.31%	79.25%

**Table 3:** Classification accuracy of each class for the Acoustic Scene Classification Datase.

Classes	Features				
	FFT-MBE	EMD-MBE	SMBE	FFT + EMD	SMBE + FFT + EMD
Airport	53.90%	40%	37.36%	40%	55.094%
Bus	52.89%	82.23%	40.9%	63.22%	76.033%
Metro	60.53%	32.18%	34.1%	51.34%	57.85%
Metro Station	57.14%	42.85%	52.5%	54.44%	54.44%
Park	70.24%	63.22%	66.11%	74.38%	65.29%
Public Square	42.12%	44.9%	33.33%	47.22%	49.07%
Shopping Mall	56.27%	64.87%	55.91%	59.50%	62.72%
Street Pedestrian	32.79%	28.34%	37.25%	39.27%	36.03%
Street Traffic	75.61%	72.76%	72.76%	76.83%	80.08%
Tram	46.74%	54.4%	50.96%	55.17%	41.37%

**Table 4:** Classification accuracy of each class for the Low Complexity ASC Dataset.

Classes	Features				
	FFT-MBE	EMD-MBE	SMBE	FFT + EMD	SMBE + FFT + EMD
Indoor	78.72%	77.87%	73.01%	81.34%	78.72%
Outdoor	81.67%	76.80%	84.54%	80.54%	82.29%
Transportation	92.83%	85.28%	79.90%	92.60%	94.15%

**Table 5:** Classification accuracy of each class for the Urbansound8k dataset.

Classes	Features				
	FFT-MBE	EMD-MBE	SMBE	FFT + EMD MBE	SMBE + FFT + EMD-MBE
air_conditioner	39.2%	41.5%	30.6%	44.9%	43.1%
car_horn	70.92%	32.38%	20.9%	74.90%	71.52%
children_playing	70.4%	50.5%	55.6%	66.5%	61.4%
dog_bark	71.3%	63.6%	66.1%	69.4%	64.2%
drilling	60.3%	60.7%	54.6%	65.6%	64.1%
engine_idle	51.49%	51.54%	40.3%	52.26%	58.61%
gun_shot	84.60%	52.80%	24.2%	76.54%	70.82%
jack_hammer	61.58%	51.73%	38.9%	60.00%	56.35%
siren	69.18%	73.35%	66%	77.14%	72.80%
street_music	78.3%	57.7%	62.3%	72%	73.7%

**Table 6:** Classification accuracy of each class for the custom dataset.

Classes	Features				
	FFT-MBE	EMD-MBE	SMBE	FFT+EMD	SMBE + FFT + EMD
Car Passing	74.24%	53.63%	65.67%	81.14%	81.48%
Rain	85.28%	81.42%	79.09%	89.33%	88.71%
Walking	61.57%	71.09%	80.52%	66.29%	71.81%
Wind	83.28%	70.57%	62.43%	78.71%	75%

## Data Availability

Not applicable.
